# Providing the best chest compression quality: Standard CPR versus chest compressions only in a bystander resuscitation model

**DOI:** 10.1371/journal.pone.0228702

**Published:** 2020-02-13

**Authors:** Bernhard Rössler, Julius Goschin, Mathias Maleczek, Felix Piringer, Rainer Thell, Martina Mittlböck, Karl Schebesta

**Affiliations:** 1 Medical Simulation and Emergency Management Research Group, University Department of Anaesthesia, Intensive Care Medicine and Pain Medicine, Medical University of Vienna, Vienna, Austria; 2 Academic Simulation Center Vienna, Medical University of Vienna and Vienna Hospital Association, Vienna, Austria; 3 St. John Ambulance, Vienna, Austria; 4 Center for Medical Statistics, Informatics, and Intelligent Systems, Medical University of Vienna, Medical University of Vienna, Vienna, Austria; Universita degli Studi di Roma La Sapienza, ITALY

## Abstract

**Aim of the study:**

Bystander-initiated basic life support (BLS) for the treatment of prehospital cardiac arrest increases survival but is frequently not performed due to fear and a lack of knowledge. A simple flowchart can improve motivation and the quality of performance. Furthermore, guidelines do recommend a chest compression (CC)-only algorithm for dispatcher-assisted bystander resuscitation, which may lead to increased fatigue and a loss of compression depth. Consequently, we wanted to test the hypothesis that CCs are more correctly delivered in a flowchart-assisted standard resuscitation algorithm than in a CC-only algorithm.

**Methods:**

With the use of a manikin model, 84 laypersons were randomized to perform either flowchart-assisted standard resuscitation or CC-only resuscitation for 5min. The primary outcome was the total number of CCs.

**Results:**

The total number of correct CCs did not significantly differ between the CC-only group and the standard group (63 [±81] vs. 79 [±86]; p = 0.394; 95% CI of difference: 21–53). The total hand-off time was significantly lower in the CC-only group than in the standard BLS group. The relative number of correct CCs (the fraction of the total number of CCs achieving 5-6cm) and the level of exhaustion after BLS did not significantly differ between the groups.

**Conclusion:**

Standard BLS did not lead to an increase in correctly delivered CCs compared to CC-only resuscitation and exhibited significantly more hand-off time. The low rate of CCs in both groups indicates the need for an increased focus on performance during BLS training.

## Introduction

Sudden cardiac arrest claims 700,000 victims each year in Europe alone.[1] Most of these sudden cardiac arrests are witnessed by lay people. Many of the victims survive because bystander-initiated cardiopulmonary resuscitation (CPR) at least doubles the survival rates of cardiac arrest.[2–4] Therefore, lay people are fundamental as a functional chain of survival.[5,6] Unfortunately, the rate of bystander CPR is still very low. This observation is attributed to the fear of making mistakes, thus harming the collapsed individual, and a reluctance to perform mouth-to-mouth ventilation, among others.[7,8]

As simple algorithms are easier to acquire and retain, the European Resuscitation Council (ERC) aimed to simplify the basic life support (BLS) algorithm and to design a sequence that would be easy to remember and apply, even for untrained lay people.[9,10] A meta-analysis demonstrated that a simplification of the algorithm (compression-only dispatcher-assisted bystander CPR) can lead to a 22% increase in survival to hospital discharge.[11] These results are similar to data from Japan, which revealed an increased number of bystander initiated CPRs and an increased survival of out of hospital cardiac arrest (OHCA) rate with favourable neurologic outcomes when chest-compressions only (CC-only) CPR was performed.[12] The increased rate of bystander-initiated BLS may be because there is no need to perform mouth-to-mouth ventilations with this method, as well as the fact that there is a reduced fear of making mistakes.[8] Furthermore, the presence of a simple flowchart indicating the necessary steps that need to be performed in BLS significantly reduces the fears of bystanders and improves the quality of BLS.[13]

To optimize outcomes, the quality of BLS is crucial. Therefore, the current ERC guidelines recommend a CC depth of 5-6cm and a CC-only algorithm for dispatcher-assisted resuscitation.[9] Nevertheless, the correct depth is often not achieved in bystander CPR after OHCA. Although improved outcomes have been reported with CC-only CPR, there is concern that CC-only CPR could lead to a more prominent decline in the compression depth over time compared to standard BLS.[11,14] Even short interruptions to CCs regardless of ventilations have been proposed to improve recuperation, thus positively impacting the compression depth.[15] Therefore, a simplified CC-only algorithm, as is currently recommended by the ERC, may be easy to remember but could lead to increased exhaustion and a loss of compression depth due to fatigue. Nevertheless, objective data are sparse. In light of the heterogeneous reports regarding the quality of CPR under a CC-only algorithm, little is known about the impact of the two algorithms that are currently recommended in regards to the optimal depth of CCs.[10]

Consequently, we tested the hypothesis that CCs are delivered at a more correct depth when utilizing the standard BLS algorithm compared to the use of the CC-only algorithm in a flowchart-assisted manikin resuscitation model.

## Materials and methods

After the approval of the Institutional Review Board of the Medical University of Vienna, Vienna, Austria (IRB Number 1136/2015), the investigation was conducted as a prospective, randomized controlled trial. Volunteers of non-medical professions (thus excluding nurses, medical doctors, physiotherapists, ergo therapists, and paramedics) who were at least 18 years of age and consisted of both males and females were enrolled (participant recruitment June 2015—April 2016). Pregnant women and people with physical impairments or illnesses that prohibited physical effort were excluded.

After providing written informed consent, the participants were randomized by using a computer-generated random sequence that was kept in opaque and sealed envelopes. A computer block randomization (6 blocks, 14 participants in each block) was performed at www.randomization.com. The participants were allocated to perform CPR according to either standard BLS or with a CC-only algorithm and were asked to undertake any action that they deemed necessary to help the person, which was simulated by a resuscitation manikin.

**Fig 1:**
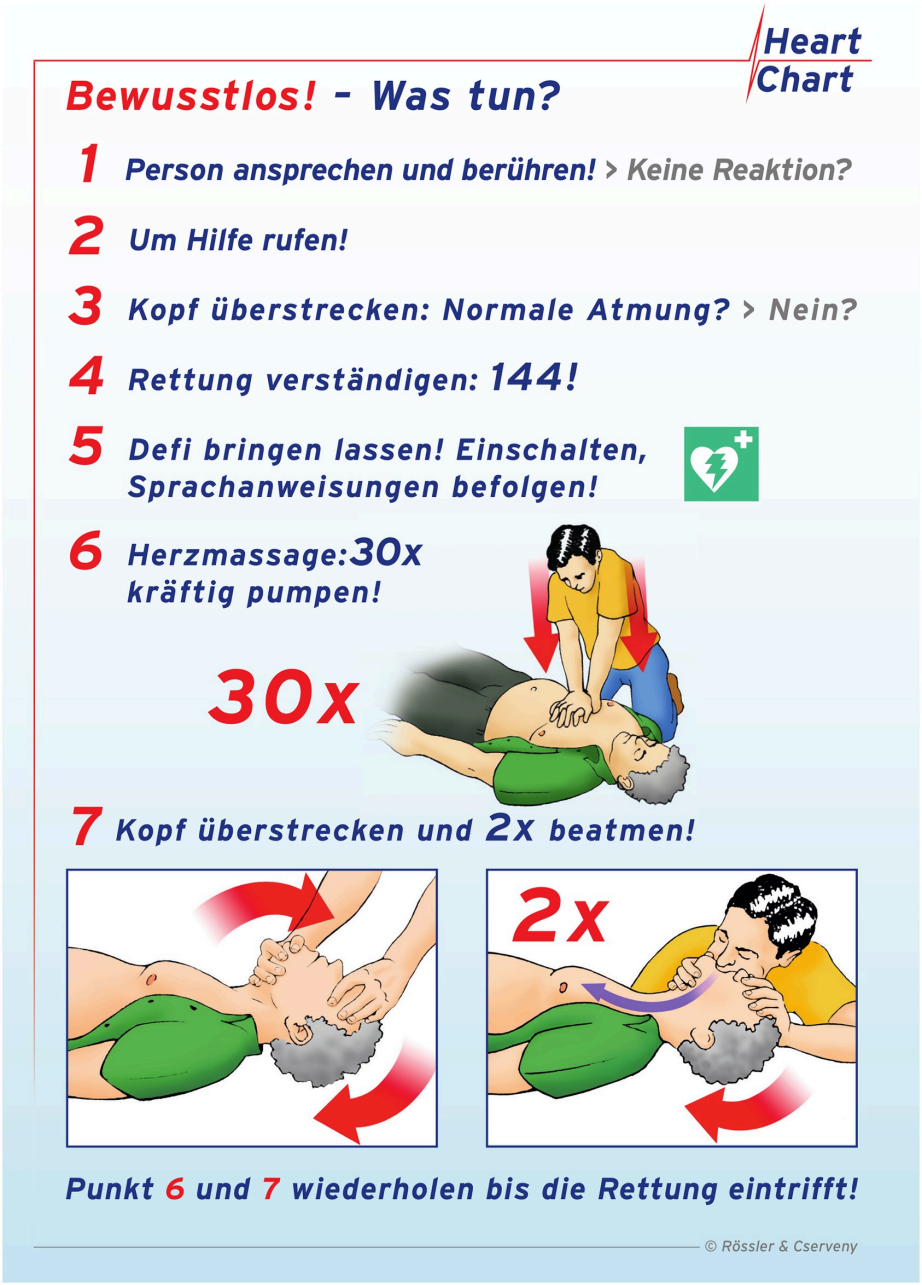
Flowchart of standard CPR. Unconscious!—What to do? 1. Speak to and touch the collapsed person! No reaction? 2. Shout for help! 3. Open airway: Breathing?—No? 4. Dial emergency medical service: 144! 5. Send for an Automated External Defibrillator: Activate and follow voice instructions! 6. Chest compressions: 30x Push hard! 7. Open airway and give two mouth-to-mouth ventilations. Repeat 6 and 7 until emergency medical service arrives.

**Fig 2:**
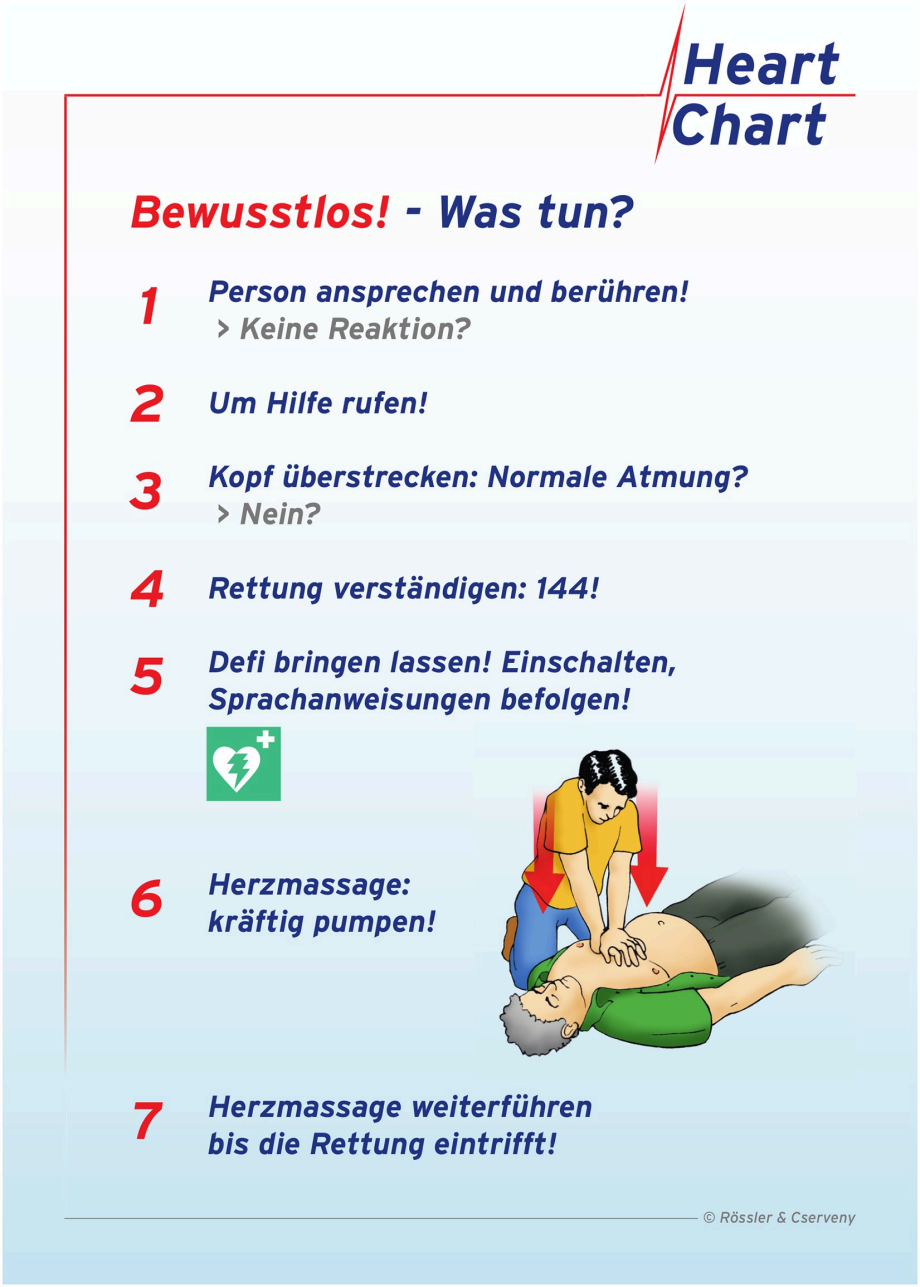
Flowchart of CC-only. Unconscious!—What to do? 1. Speak to and touch the collapsed person! No reaction? 2. Shout for help! 3. Open airway: Breathing?—No? 4. Dial emergency medical service: 144! 5. Send for an Automated External Defibrillator: Activate and follow voice instructions! 6. Chest compressions: Push hard in the middle of the chest. Continue until emergency medical service arrives.

Both flowcharts led the participants through the initial steps of the CPR algorithm (including the identification of a victim in cardiac arrest and the activation of the emergency medical services) identically. The BLS flowchart imposed 30 CCs to be delivered beside a matching pictogram (“30x push hard”). Thereafter, the helper should “open airway and give two mouth-to-mouth ventilations”. The written information on rescue breaths was again supported by two additional pictograms pointing the helper to open the airway correctly before applying two ventilation breaths (Fig 1). The CC only flowchart stated “push hard in the middle of the chest”, supported by a similar pictogram regarding chest compressions as in the standard BLS algorithm, but naturally no information on rescue breaths was provided (Fig 2).

At the beginning of the scenario, the manikin was positioned in a supine position on the floor of a room that was previously prepared to minimize outside interruptions. The participants in both groups immediately received a flowchart depicting the CPR algorithms (BLS, see Fig 1 or CC-only, see Fig 2) at the beginning of the 5min scenario without further instructions regarding its content. The participants were instructed to verbally indicate the subjective decline in the resuscitation quality without stopping CPR, but they did not receive further instructions or support during the CPR session. While participating in the scenario, the participants were blinded to the elapsed time. The data of the steps that were performed, as well as the steps that were potentially omitted and the exact times, were electronically documented and stored on hardcopy case report forms, when appropriate.

### Endpoints

The primary endpoint consisted of the total number of CCs that achieved the correct depth of 5-6cm, as is recommended by the ERC and American Heart Association, in 5min of manikin CPR.[10,16] The authors hypothesized that CCs are more correctly delivered at 5-6cm when utilizing the standard BLS algorithm compared to the use of the CC-only algorithm in a flowchart-assisted manikin resuscitation model.

The secondary endpoints included the (i) hand-off time (HOT), which was defined as the sum of the total time in which no CCs were provided within 5min of the observation phase; (ii) time to the administration of CCs; (iii) total number of CCs; (iv) relative number of correct CCs (%), (v) CCs >5cm; (vi) relative number of CCs >5cm; and (vii) average compression rate. The time to the commencement of CCs, as well as all interruptions of the CCs (e.g., ventilations or pauses), were included in the HOT calculations.

Furthermore, the points in time when the participants subjectively felt a loss in the quality of CPR due to fatigue were evaluated. Additionally, the levels of confidence and exhaustion were rated by the participants at the end of the 5min scenario on a 10-item Likert-like scale (1 = no exhaustion at all, 10 = utmost exhaustion).[17] If a participant chose to abort the resuscitation attempts, then the open-ended question of “Why did you discontinue the resuscitation attempts?” was raised.

### Statistical methods

#### Sample size

To detect a clinically important difference of 20% in the compression depth with a power of 0.8 and a significance level set at 0.05, the sample size calculation yielded a total necessary number of participants of 74. To account for a dropout rate of approximately 10%, 84 participants were recruited. The data regarding the compression depth with the utilization of standard CPR techniques in a manikin model (43±12mm) were provided by a previous publication and were used to estimate the sample size.[13]

The data are described as absolute frequencies and percentages for the categorical data and by using means and standard deviations (SDs) or 95% confidence intervals, where appropriate. The analysis was performed as the intention to treat. All tests for the p-values were two-sided, and p≤0.05 was regarded to be statistically significant. Student’s t-test and Fisher-Yates test were used as appropriate. The results of the Likert-like scales were treated as interval-measures, as is common practice. Thus, these results were analysed by using parametric tests.[18,19] No corrections for multiple testing were performed, as the secondary outcome parameters are hypothesis generating only. The evaluation of the performance data was performed by an independent investigator by using a computer attached Laerdal Skill Reporting System with the Segstats software (Version 2.3.0, Laerdal Medical, Stavanger, Norway). Local data management was performed by using Microsoft Excel for Mac (Version 14.1.0, Microsoft Corporation, Redmond, WA) and SPSS Statistics (version 22, IBM Corporation, Armonk, NY). The graphics were created with Prism 5 for Mac OS X (Version 5.0a, GraphPad Software Inc., La Jolla, CA).

## Results

**Fig 3:**
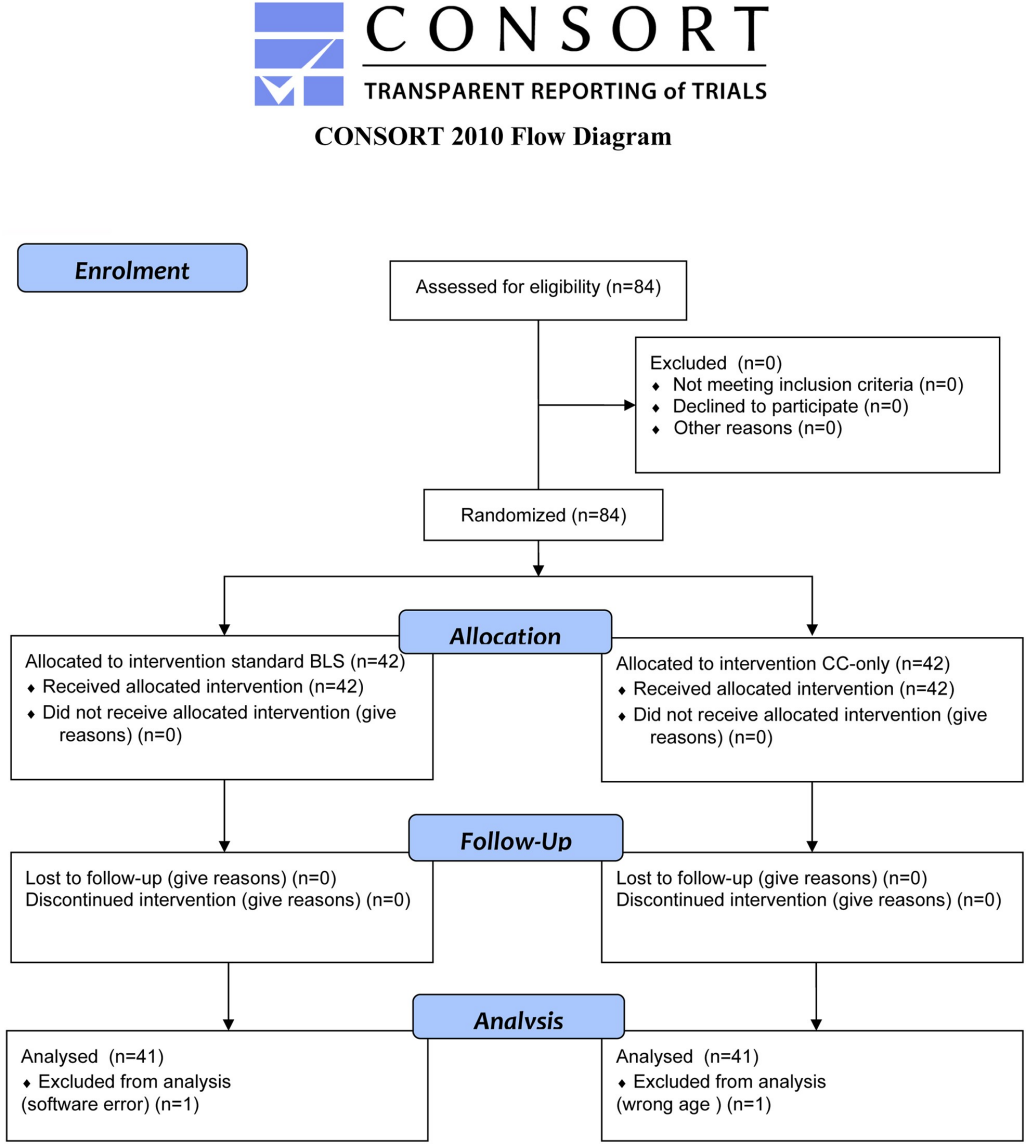
Flow diagram of study participants.

A total of 84 participants were randomized to participate in the trial. One dataset in the standard BLS group was lost due to a software error. One individual in the CC-only group was excluded due to the precise age information not being correctly provided prior to the randomization. Forty-one participants completed the scenario in each group. The demographic data are presented in Table 1. Study enrolment is presented in Fig 3.

**Table 1:** Demographic data of the participants; means (±SDs) or absolute number (%).

	CC-only n = 41	BLS n = 41
Age (y)	26.8 (±10.5)	29.1 (±11.4)
Female	17 (41%)	23 (56%
Would perform CPR in actual patient (yes)	36 (±88)	36 (±88)
Has performed CPR in actual patient (yes)	1 (±2)	2 (±5)
Has participated in the BLS course (yes)	18 (±44)	21(±51)
Month to last BLS course	29 (±36)	38 (±80)

CC-only: chest compression only; BLS: standard basic life support.

The total number of correct CCs (5-6cm) was 79 (±86) in the standard BLS group and 63 (±81) in the CC-only group (p = 0.394; 95% CI of difference: -21-53). The total HOT was 72 (±56)s in the CC-only group vs. 130 (±38)s in the standard BLS group (p<0.001; 95% CI of difference: 37–79). The time to commencement of the CCs was 53 (±26)s in the standard BLS group and 51 (±25)s in the CC-only group (p = 0.762; 95% CI of difference: -10-13). The total number of CCs was 395 (±152) in the CC-only group and 278 (±84) in the standard BLS group (p<0.001; 95% CI of difference: -171 to 63). Further details on quality of CCs in both groups are presented in Table 2. No participants in either group discontinued CPR before the end of the predefined 5min scenario duration. The time to exhaustion, the level of exhaustion at the end of the resuscitation efforts and the confidence level are provided in Table 2.

**Table 2:** Outcome Data; means (±SDs) or absolute number (%); 95% CI of difference.

	CC-only n = 41	BLS n = 41	p	95% CI of Difference
Time to exhaustion (s)	171 (±66)	201 (±54)	0.189	-15-74
Level of exhaustion	4.8 (±2.4)	4.6 (±2.5)	0.656	-1.3–0.8
Level of confidence	6.0 (±2.6)	5.9 (±2.5)	0.896	-1.2–1.0
Average depth of CC	45 (±11)	47 (±13)	0.310	-3 to +8
Number of CCs at 5-6cm (%)	18 (±23)	29 (±29)	0.078	-1 to 22
Number of CCs >5cm	123 (±156)	151 (±142)	0.398	-37 to 93
Number of CCs >5cm (%)	34 (±40)	51 (±43)	0.060	-1 to 36
CC rate per minute	103 (±25)	102 (±21)	0.729	-12 to 8
Incomplete recoil of CC (%)	31(±40)	34(±37)	0.620	-14 to 20

Table 2 provides details on the secondary outcome parameters. Data are presented as the means (±SDs). The level of confidence and exhaustion were evaluated on a Likert-like scale (1–10, 1 indicating no exhaustion/confidence at all, whereas 10 indicates the utmost exhaustion/confidence).

## Discussion

This randomized, controlled simulation trial demonstrated that the total number of correct CCs did not significantly differ when flowchart-assisted CPR was performed by untrained lay persons using a standard BLS model or a CC-only algorithm. Our primary hypothesis that CCs are more correctly delivered when utilizing standard BLS compared to the CC-only algorithm was therefore rejected. Furthermore, we could not demonstrate any differences in the time to fatigue or the level of exhaustion between the intervention groups within 5min of performing BLS.

To improve the resuscitation quality, CC-only CPR has been recommended by an international consensus for dispatcher-assisted bystander CPR efforts and for untrained helpers.[10] However, these results are in contrast to the findings of Heidenreich et al.[20] In their 9min resuscitation model, CC-only CPR was performed by students and resulted in significantly more correct CCs than the use of standard BLS. Nevertheless, in 2012, the same authors published a trial wherein elderly participants performed CC-only vs. standard BLS, and they observed that standard BLS resulted in a greater number of adequate compressions in all but the first minute of resuscitation.[14] The results of our trial do not support either of the aforementioned, conflicting results, but they do demonstrate that the total number of correct CCs and the time to exhaustion were similar in both study groups.

The introduction of a dual dispatch system, including first responders and emergency medical service activation, can lead to a significant decrease in the time from the call to the arrival of professional help in OHCA.[21,22] To recreate the current response times in urban two-tier systems, this trial was performed utilizing a 5min simulation cardiac arrest scenario.

In both trials that were conducted by Heidenreich et al, adequate CC depth was defined at >3.8cm, which was significantly lower than the current recommended guidelines; thus, this depth was lower than the threshold that was used in the current trial. Additionally, in both of the aforementioned studies, the participants were narrowly defined groups (students vs. elderly individuals).

Theoretically, the number of correct CCs in the use of CC-only CPR should be significantly higher than in the use of standard CPR; however, a positive impact of interruptions of CCs, regardless of rescue breaths, has been described. Min and colleagues compared a group that performed CPR with defined breaks to a group that recuperated during the CCs.[15] They concluded that the group that had 10 second breaks after performing 100 CCs had the highest number of adequate CCs. In contrast to the study by Heidenreich et al, the compression depth in the trial by Min was defined as >5cm. Additionally, Min and colleagues did not include a standard-BLS group and conducted it in a group of paramedic trainees, which may have resulted in a significant selection bias and may not reflect the performance expected by lay bystanders.

Although Min et al included an introductory BLS training regimen prior to the data collection (to improve standardized performance), this potentially created a bias of overestimating the quality of the BLS. The skill levels of lay helpers rapidly decline after CPR courses, and the true effects at a later point in time are potentially much smaller. Nonetheless, in times of ubiquitous access to cognitive aids (e.g. through the use of smartphones), the current trial deliberately omitted testing after a BLS course, but provided the helper with a cognitive aid that resembles the potential support that is rapidly available at the time of collapse. In comparison to the previous trials, this situation allowed for a more realistic evaluation of the non-medical helpers’ performances.

As expected, and due to the lack of interruptions for the rescue breaths, this trial demonstrated a significantly higher total amount of CCs in the CC-only group. The CC- rate was adequate in both groups, nevertheless. However, the higher number of CCs in the CC-only group did not result in higher accuracy of compression depth. In contrast, the relative number of correct CCs exhibited a statistically insignificant trend in favour of the standard BLS. Nevertheless, the low rate of correct depth in both groups require stronger emphasis in future BLS training.

In contrast to previously published data, work by Stiell and colleagues identified a peak in survival at 4.6cm, which is a value that is below the currently recommended compression depth of 5-6cm.[23,24] Consequently, it remains to be evaluated as to whether increased number of CCs in the CC-only group outweighs the trend of the decreased fraction of the currently recommended correct depth of 5-6cm. Furthermore, the data from an observational trial indicated an increased risk of injury in chest compressions >6cm.[25]

Since the expected degree of exhaustion increases with longer CPR durations, CPR providers should change approximately every 2min.[10] The loss of quality is more evident in the uses of CC-only or CPR with very limited interruptions.[15,17] Interestingly, in the current trial, the time interval to the self-reported point of exhaustion did not significantly differ between the groups and occurred at approximately 3min. Neither did the level of exhaustion after 5min of CPR. Although the timeframe of the scenario was limited to represent the response intervals of an urban first-responder system, these intervals were potentially too short to lead to subjective tiring or exhaustion resulting in the discontinuation of CPR.[21] Nevertheless, while two tier systems in cities can help to achieve shortened intervals of bystander CPR requirements, these intervals can be significantly longer in rural settings.

Although the introduction of a cognitive aid does increase the helper’s confidence, little is known concerning how much content can be conveyed without affecting the time to CCs or without overwhelming the helper, thus further reducing confidence.[13] In this trial, both groups received a flowchart depicting the respective CPR algorithms at the beginning of the scenario. The level of confidence was high, and no significant difference was detected in either of the groups. Thus, both flowcharts can be adequately utilized as cognitive aids ‘on the spot’ without prior introduction in OHCA situations.

## Limitations

This trial was conducted in a manikin-based, cardiac arrest simulation. Therefore, the direct translation of the manikin-derived data into clinical practice may be limited. However, manikins provide an acceptable setting for analysing the quality of CCs during CPR.[17,26,27] Additionally, the simulated setting may have influenced the willingness to perform CPR, as well as the fears of the lay people. Nevertheless, previous publications have reported emotional involvements in manikin trials to be comparable to real-life emergency situations.[28] As in a previous publication, the flowchart was immediately provided to the participant.[13] This can potentially influence the impacts of the chart. In real-life settings, a flowchart or an app may be studied in advance; in contrast, by immediately providing the chart at the beginning of the scenario, the positive impacts on CPR quality can be underestimated (specifically, the time to start commencing CCs). The interval studied was chosen to represent the setting in a large urban area with an effective first responder system. Nevertheless, in many rural areas response times can be significantly higher than the studied interval. Finally, most of the recruitment was conducted in a BLS course centre prior to the start of the BLS course. Although the impact of knowledge from the course on the study can be ruled out, a motivation bias, as well as a selection bias in both groups for younger and potentially fitter individuals may have affected the results.

## Conclusion

In this five-minute resuscitation scenario and in a sample of a general lay population, the number of correct CCs that were produced did not significantly differ between the CC-only and the standard BLS groups. Nevertheless, the total number of CCs was significantly higher and the HOT was reduced in the CC-only group, but the majority of the CCs were of insufficient compression depths. The benefit of CC-only BLS diminished when considering the number of correctly performed CCs. The time to exhaustion did not significantly differ, thus concurring with the current recommendations. The low rate of correct CC depths in both groups indicates a need for an increased emphasis on high-quality CCs to optimize the compression depths during BLS training.

## Supporting information

S1 Checklist CONSORT 2010 checklist of information to include when reporting a randomised trial*. (DOC)

S1 Protocol (PDF)

## References

[1] Sans S, Kesteloot H, Kromhout D. The burden of cardiovascular diseases mortality in Europe. Task Force of the European Society of Cardiology on Cardiovascular Mortality and Morbidity Statistics in Europe. Eur Heart J 1997;18:1231–48. 9508543

[2] Hasselqvist-Ax I, Riva G, Herlitz J, et al Early Cardiopulmonary Resuscitation in Out-of-Hospital Cardiac Arrest. N Engl J Med 2015. 10.1056/NEJMoa1405796 26061835

[3] Larsen MP, Eisenberg MS, Cummins RO, Hallstrom AP. Predicting survival from out-of-hospital cardiac arrest: A graphic model. Ann Emerg Med 1993. 10.1016/s0196-0644(05)81302-2 8214853

[4] Nolan J. Cardiac Arrest and Cardiopulmonary Resuscitation. Semin Neurol 2017;37:005–12. 10.1055/s-0036-1597832 28147412

[5] Müller D, Agrawal R, Arntz HR. How sudden is sudden cardiac death? Circulation 2006. 10.1161/CIRCULATIONAHA.106.616318 16952983

[6] van Alem AP, Vrenken RH, de Vos R, Tijssen JGP, Koster RW. Use of automated external defibrillator by first responders in out of hospital cardiac arrest: prospective controlled trial. BMJ 2003;327:1312–0. 10.1136/bmj.327.7427.1312 14656837 PMC286314

[7] Swor R, Khan I, Domeier R, Honeycutt L, Chu K, Compton S. CPR Training and CPR Performance: Do CPR-trained Bystanders Perform CPR? Acad Emerg Med 2006;13:596–601. 10.1197/j.aem.2005.12.021 16614455

[8] Savastano S, Vanni V. Cardiopulmonary resuscitation in real life: The most frequent fears of lay rescuers. Resuscitation 2011;82:568–71. 10.1016/j.resuscitation.2010.12.010 21333434

[9] Koster RW, Baubin MA, Bossaert LL, et al European Resuscitation Council Guidelines for Resuscitation 2010 Section 2. Adult basic life support and use of automated external defibrillators. Resuscitation 2010;81:1277–92. 10.1016/j.resuscitation.2010.08.009 20956051 PMC7116923

[10] Perkins GD, Handley AJ, Koster RW, et al European Resuscitation Council Guidelines for Resuscitation 2015. Section 2. Adult basic life support and automated external defibrillation. Resuscitation 2015. 10.1016/j.resuscitation.2015.07.015 26477420

[11] Hüpfl M, Selig HF, Nagele P. Chest-compression-only versus standard cardiopulmonary resuscitation: A meta-analysis. Lancet 2010. 10.1016/S0140-6736(10)61454-7 PMC2987687 20951422

[12] Iwami T, Kitamura T, Kiyohara K, Kawamura T. Dissemination of chest compression-only cardiopulmonary resuscitation and survival after out-of-hospital cardiac arrest. Circulation 2015. 10.1161/CIRCULATIONAHA.114.014905 26048093

[13] Rossler B, Ziegler M, Hupfl M, Fleischhackl R, Krychtiuk KA, Schebesta K. Can a flowchart improve the quality of bystander cardiopulmonary resuscitation? Resuscitation 2013;84:982–6. 10.1016/j.resuscitation.2013.01.001 23306815

[14] Heidenreich JW, Bonner A, Sanders AB. Rescuer fatigue in the elderly: Standard vs. Hands-only CPR. J Emerg Med 2012. 10.1016/j.jemermed.2010.05.019 20634016

[15] Min MK, Yeom SR, Ryu JH, et al A 10-s rest improves chest compression quality during hands-only cardiopulmonary resuscitation: A prospective, randomized crossover study using a manikin model. Resuscitation 2013. 10.1016/j.resuscitation.2013.01.035 23402967

[16] Kleinman ME, Brennan EE, Goldberger ZD, et al Part 5: Adult Basic Life Support and Cardiopulmonary Resuscitation Quality. Circulation 2015;132:S414–35. 10.1161/CIR.0000000000000259 26472993

[17] McDonald CH, Heggie J, Jones CM, Thorne CJ, Hulme J. Rescuer fatigue under the 2010 ERC guidelines, and its effect on cardiopulmonary resuscitation (CPR) performance. Emerg Med J 2013. 10.1136/emermed-2012-201610 22851670

[18] Norman G. Likert scales, levels of measurement and the “laws” of statistics. Adv Health Sci Educ Theory Pract 2010;15:625–32. 10.1007/s10459-010-9222-y 20146096

[19] Carifio J, Perla R. Resolving the 50-year debate around using and misusing Likert scales. Med Educ 2008;42:1150–2. 10.1111/j.1365-2923.2008.03172.x 19120943

[20] Heidenreich JW, Berg RA, Higdon TA, Ewy GA, Kern KB, Sanders AB. Rescuer Fatigue: Standard versus Continuous Chest-Compression Cardiopulmonary Resuscitation. Acad Emerg Med 2006. 10.1197/j.aem.2006.06.049 17015418

[21] Saner H, Morger C, Eser P, von Planta M. Dual dispatch early defibrillation in out-of-hospital cardiac arrest in a mixed urban–rural population. Resuscitation 2013;84:1197–202. 10.1016/j.resuscitation.2013.02.023 23518012

[22] Zijlstra JA, Stieglis R, Riedijk F, Smeekes M, van der Worp WE, Koster RW. Local lay rescuers with AEDs, alerted by text messages, contribute to early defibrillation in a Dutch out-of-hospital cardiac arrest dispatch system. Resuscitation 2014;85:1444–9. 10.1016/j.resuscitation.2014.07.020 25132473

[23] Stiell IG, Brown SP, Nichol G, et al What Is the Optimal Chest Compression Depth During Out-of-Hospital Cardiac Arrest Resuscitation of Adult Patients? Circulation 2014;130:1962–70. 10.1161/CIRCULATIONAHA.114.008671 25252721

[24] Stiell IG, Brown SP, Christenson J, et al What is the role of chest compression depth during out-of-hospital cardiac arrest resuscitation?. Crit Care Med 2012;40:1192–8. 10.1097/CCM.0b013e31823bc8bb 22202708 PMC3307954

[25] Hellevuo H, Sainio M, Nevalainen R, et al Deeper chest compression—More complications for cardiac arrest patients? Resuscitation 2013. 10.1016/j.resuscitation.2013.02.015 23474390

[26] Yamanaka S, Huh JY, Nishiyama K, Hayashi H. The optimal number of personnel for good quality of chest compressions: A prospective randomized parallel manikin trial. PLoS One 2017;12:e0189412 10.1371/journal.pone.0189412 29267300 PMC5739419

[27] Song Y, Chee Y, Oh J, Ahn C, Lim TH. Smartwatches as chest compression feedback devices: A feasibility study. Resuscitation 2016;103:20–3. 10.1016/j.resuscitation.2016.03.014 27004719

[28] Hunziker S, Laschinger L, Portmann-Schwarz S, Semmer NK, Tschan F, Marsch S. Perceived stress and team performance during a simulated resuscitation. Intensive Care Med 2011;37:1473–9. 10.1007/s00134-011-2277-2 21695475

