# Supplementary material for: Providing the best chest compression quality: Standard CPR versus chest compressions only in a bystander resuscitation model
Source: PLoS One. 2020 Feb 13;15(2):e0228702. doi: 10.1371/journal.pone.0228702 (PMC7017996; doi:10.1371/journal.pone.0228702)
Supplement: S1 Protocol — (PDF) [file pone.0228702.s002.pdf]

Medical University of Vienna

Department of Anaesthesia, General Intensive Care and Pain Management

Allgemeines Krankenhaus der Stadt Wien

Währinger Gürtel 18-20

A-1090 Wien, Austria

Tel +43 1 40400 41000

Fax +43 1 40400 68250

**STUDY PROTOCOL**

# Flowchart-Assisted Resuscitation.

# Standard CPR vs. Chest Compressions only:

# What happened to the Quality?

**Academic investigators:** Priv.-Doz. Dr. Bernhard Rössler, MIH

Assoc. Prof. Dr. Karl Schebesta,

Dr. Rainer Thell, St. John Ambulance

**Statistician:** Ao.Univ.-Prof. Mag. Dr. Martina Mittlböck

**Principle investigator:** Priv.-Doz. Dr. Bernhard Rössler, MIH

**Senior author, corresponding author:** Assoc. Prof. Dr. Karl Schebesta

**Sponsor:** Medical University of Vienna, Department of Anaesthesia, General Intensive Care and Pain Management, Währinger Gürtel 18-20, 1090 Vienna, Austria

**<u>Ethics Board Timeline:</u>** **11.02.2015 Submission Deadline**

**10.03.2015 Meeting**

## ABSTRACT

**Background:**

More than two thirds of sudden cardiac arrests are witnessed by bystanders. Bystander cardiopulmonary resuscitation (CPR) doubles survival from cardiac arrest. Importantly, even in witnessed cardiac arrests only 20% of the bystanders commence basic life support. Common reasons for not commencing include panicking and the perceived inability to perform CPR correctly. A meta-analysis could demonstrate that a simplification of the algorithm (compression-only dispatcher-assisted bystander CPR) led to a 22% increase in survival-to-hospital discharge. A recently published trial of the research group could demonstrate that the presence of a flowchart has a positive effect on the quality of BLS while at the same time increasing the rescuers' confidence. Nonetheless, performing CPR is exhausting. Previous publications have emphasized increasing fatigue with the duration of CPR efforts in both, standard and chest compressions-only CPR. Consequently, we wanted to test the hypothesis that chest compressions (CC) are delivered more correctly regarding the depth when utilizing the standard BLS algorithm with the aid of a flowchart as compared to the CC only algorithm utilizing an adapted CPR flowchart in a manikin resuscitation model.

**Methods:**

After consent of the Research Ethics Board of Medical University of Vienna and obtaining written informed consent of the participants, 84 medically untrained laypersons will be randomised to perform flow-chart assisted CPR for 300s following standard CPR guidelines or CC only CPR. The primary outcome parameter will be the total number of CC achieving the correct depth of 50-60mm. Secondary outcome parameters will be hands-off time, the total number of CC, and the compression rate. The total number of delivered rescue breaths, tidal volume, and time to deliver these will also be evaluated. Furthermore, the subjective point of exhaustion affecting the CPR quality, the reason for discontinuation of CPR if stopped within the 300sec. period and the exhaustion at the end of the CPR measures will be evaluated.

**Background:**

Since the introduction of the modern Basic Life Support (BLS) in the 1950, on-going efforts have been made to improve the quality of first aid measures [**Handley** *et al.* 2005, **Koster** *et al.* 2010]. International BLS algorithms have been designed, validated and published to meliorate the outcome after a cardiac arrest [**Koster** *et al.* 2010].

In many European countries cardiac arrest and sudden death due to coronary ischaemia is the primary single cause of death in adults. Across Europe, with a total population of approx. 830 Million inhabitants, emergency medical service treated cardiac arrest occurs in a range of 17-53/1000,000/year [**Gräsner** *et al.* 2013].

Many of these victims of sudden cardiac arrest could survive if bystanders would act immediately while shock-able heart rhythms are still present, but successful resuscitation is unlikely once the rhythm has deteriorated to asystole [**Valenzuela** *et al.* 1998].

Chances of survival from witnessed ventricular fibrillation decreases by 7-10% every minute without cardiopulmonary resuscitation (CPR) [**Valenzuela** *et al.* 1998]. In urban areas, the Emergency Medical Services response interval is eight minutes or more [**vanAlemAP** *at al.* 2003]. Overall, bystander CPR doubles or triples survival from witnessed cardiac arrest [**Wissenberg** *et al.* 2013].

In most cases the fundament of a functional chain of survival is lay people who may or may not have been trained in BLS courses. Especially as more than two thirds of the sudden cardiac arrests are witnessed by bystanders [**Müller** *at al.* 2006]. Unfortunately, the frequency of initiating CPR by lay people is still seen to be very low. Reasons for this fact are among others, fear of making mistakes and thus harming the collapsed or reluctance to perform mouth-to-mouth ventilation [**Swor** et al. 2006, **Svastano** et al. 2011].

Evidence shows that skill acquisition and retention is, besides regular application of the algorithm, added by the simplification of BLS sequence [***Koster*** *et al.* 2010]. In the process of improving the resuscitation guidelines it was one of the aims of the European Resuscitation Council to simplify the BLS algorithm and design a sequence that would be easy to remember and apply. A recently published trial of the research group could demonstrate that the presence of a flowchart has an additional positive effect on the quality of BLS [**Rössler** *et al.* 2013].

A meta-analysis of three previously conducted trials could demonstrate that a simplification of the algorithm (compression-only dispatcher-assisted bystander CPR) led to a 22% increase in survival-to-hospital discharge [**Hüpfl** *et al.* 2010]. These findings were so convincing that they found representation in a scientific statement of the AHA regarding pre-arrival instruction by the dispatcher [**Lerner** *et al.* 2012].

Nonetheless, even when bystanders commence CPR, publications have demonstrated fatigue in both, standard and chest compressions-only CPR [**Heidenreich** *et al.* 2012, **McDonald** *et al.* 2013]. Despite the fact that chest compressions-only CPR lead to an increase in total number of delivered chest compressions (CC), the fraction of effective CC (reaching the recommended depth) declines among elderlies with the longer duration of CPR [**Heidenreich** *et al.* 2012]. Noteworthy, in a publication by McDonald and colleagues, fatigue did not affect the rate of compressions. Nevertheless, the compression depth was affected from the second minute onwards [**McDonald** *et al.* 2013]

This fact is of clinical relevance since for every 5mm increase in compression depth in out of hospital cardiac arrests, the odds of survival and survival with a functional outcome increase [**Vadeboncoeur** *et al.* 2014]. Even though the optimal depth remains disputed, the current ERC Guidelines recommend a depth of 50-60mm [**Koster** *et al.* 2010]. Furthermore, and a depth of >50mm has been shown to be associated with improved outcome as well as functional neurological outcome [**Vadeboncoeur** *et al.* 2014]. Nevertheless, the optimal depth is not undisputed since contrary to previously published data, a recent publication by Stiell and colleagues identified the peak in survival at 45.6mm, below the currently recommended depth [**Stiell** *et al.* 2014].

The guidelines also recommend a CC rate of 100-120/min [**Koster** *et al.* 2010]. This is in line with recently published data indicating that the maximum rate of survival to hospital discharge can be achieved when compressing at 100-120/min [**Idris** *et al.* 2015]. An increase in CC rate over 120/min can decline the probability of return of spontaneous circulation possibly due to a concordant reduction in compression depth [**Monsieurs** *et al.* 2012, **Stiell** *et al.* 2012].

Consequently, we wanted to test the hypothesis that chest compressions are delivered more correctly regarding the depth when utilizing the standard BLS algorithm with the aid of a flowchart as compared to the CC only algorithm utilizing an adapted BLS flowchart in a manikin resuscitation model.

**Study Design**

The investigation will be conducted as a prospective, randomized controlled study performed by the Department of Anaesthesia, General Intensive Care and Pain Management, Medical University of Vienna, Austria, according to the Good Scientific Practise guidelines of the Medical University of Vienna. The data collection will be conducted in cooperation with the Training Centre of St. John Ambulance, Vienna, Austria.

**Study Population**

Eligible are all volunteers of ≥18 years of age of non-medical profession, irrespective of gender. Exclusion criteria are professional medical training (nurses, medical doctors, physiotherapists, ergotherepeuts, emergency medical technician or equivalent), pregnancy (as by verbal response at the time of recruitment), and parallel participation in a clinical trial, or physical impairment or illness prohibiting physical effort.

**Withdrawal and replacement of subjects**

Subjects must be withdrawn under the following circumstances:

-at their own request

-if the subject violates the conditions laid out in the consent form/information sheet or disregards instructions by the study personnel

In all cases, the reasons why subjects are withdrawn must be recorded in detail in the case report form. Should the study be discontinued prematurely, all study materials (completed, partially completed and empty case report forms) will be retained.

Subjects who do not complete the study according to protocol will be replaced. The data from the replaced subjects will be eligible for analysis of the conducted trial periods and for safety variables.

**Methodology**

**Sample Size**

In order to detect a clinically important difference of 20% in compression depth with a power of 0.8 and a significance level set at 0.05, the sample size calculation yielded a total needed number of participants at 74. Since a drop out rate of approx. 10% must be expected 84 participants will be recruited. Data regarding compression depth utilizing standard CPR techniques in a manikin model (43±12mm) were provided by a previous publication and used to estimate the sample size [**Rössler** *et al.* 2013]

**Statistical Methods**

Outcome parameters:

The primary outcome is total number of CC achieving the correct depth of 50-60mm in five minutes of manikin CPR. With this the authors want to test the hypothesis that chest compressions are delivered more correctly regarding the depth when utilizing the standard BLS algorithm with the aid of a flowchart as compared to the CC only algorithm utilizing an adapted BLS flowchart in a manikin resuscitation model. The secondary outcome parameter is hands-off time (HOT), which is defined as the sum of total time elapsed during the 300sec. of BLS in which no chest compressions are provided. This includes the pre-compression interval where the initial steps of BLS are performed as well as all interruptions of the CC, e.g. in order to ventilate or pause due to any other reason including exhaustion. Furthermore, the total number of CC, and the compression rate will be evaluated. The total number of delivered rescue breaths, tidal volume, and time to deliver these will also be evaluated.

Furthermore, questions regarding following points will be raised: “During CPR and without stopping your actions, please tell us “NOW” when you perceive your fatigue is affecting the quality of the life support performed”, “How exhausted are you now (question raised directly after discontinuing the CPR) [**McDonald** *et al.* 2013] (Lickert Like Scale 1-10). If a participant choses to abort resuscitation attempts the open ended question “Why did you discontinue the resuscitation attempts?” will be raised.

Data management and calculations

Data will be described as absolute frequencies and percentages for categorical data and using mean and standard deviations (SD) for normally distributed data. All tests for p-values are two-sided and p≤0.05 will be regarded to be statistically significant. Students t-test and chi-sqare test will be used as appropriate. As it is common practice, results of Likert-like scales will be treated as interval-measures and thereby analysed by using parametric tests [**Norman** *et al.* 2010, **Cariio** *et al.* 2008]. Local data management will be done using Microsoft Excel for Mac (Microsoft Corporation, USA) and R for Mac (R Foundation for Statistical Computing, Vienna, Austria) for statistical analysis. [**R_Development_Core_Team** 2009] Graphics will be created using PraphPad Prism for Mac (GraphPad Software Inc., La Jolla, USA).

**Description of study days**

Volunteers will be invited to participate in the trial by the study personnel. Recruitment will be conducted at a Training Centre of St. John ambulance, Vienna, Austria, before the starts of the lessons. After giving written informed consent, participants will be randomized using a web based randomization program

(www.random.org) and allocated to perform standard CPR or CC only. The randomization for group allocation will be kept in opaque and sealed envelopes.

The evaluation will be performed with an independent investigator using a computer attached Resusci-Anne Skillreporter by Laerdal-Medical® and the Laerdal Skillreporter Software with Segstat (Version 2.3.0, Laerdal Medical, Stavanger, Norway).

The participants will be asked to perform BLS for five minutes on a manikin. The participants will then be asked to undertake any action they deem necessary to rescue the person simulated by the resuscitation manikin. Participants will not be informed about the underlying hypothesis or the outcome parameters. The room will be prepared to minimise outside interruptions.

Additionally, participants will be instructed to indicate the point in time during CPR when they feel that their fatigue is affecting the quality of the life support performed. ("During CPR and without stopping your actions, please tell us "NOW" when you perceive your fatigue is affecting the quality of the life support performed.)

Participants in both groups will receive one of the charts right at the beginning of the scenario with the information that "this chart will provide information on how to perform CPR" and with the start of the clock further instructions regarding is content. Both groups will not receive any further introduction or support. At the beginning of the scenario, the manikin will be positioned in a supine position on the floor. There will be no clock visible for the participant while performing CPR. An investigator is acting as bystander able to call the emergency medical service or to be sent to look for an automated external defibrillator (which will not be available in the scenario). Nonetheless, the investigator is instructed not to provide any information on how to perform CPR and does not physically take part in the primary check, CC, or mouth-to-mouth ventilations.

Data of steps performed or left out will be documented in hardcopy on the case report form by an investigator and the exact times and details of chest compressions and ventilations will be documented electronically (Laerdal Skillreporter Software with Segstat (Version 2.3.0, Laerdal Medical, Stavanger, Norway)). Checklists of necessary steps will be created based on the ERC BLS Guidelines 2010 in the same manner as published previously [**Roessler** *et al.* 2007, **Koster** *et al.* 2010].

After the five-minute period has ended, the participants will be informed that the emergency medical service is now taking over and that they can stop performing CPR. Directly thereafter, they will be asked the following questions "How exhausted are you now on a scale from 1 to 10, where 1 indicates no exhaustion at all and 10

maximum exhaustion?" (Lickert Like 1-10). If a participant choses to abort resuscitation attempts before the five minutes are over, the open-ended question "Why did you discontinue the resuscitation attempts?" will be raised.

**Risk/benefit assessment:**

Apart from physical strain such as muscle pain or abrasion on the palm of the hand or knee after delivering chest compressions, there is no direct risk as the consequence of participation in the trial. To ensure participants added value, detailed information about the ERC CPR algorithm will be given after data collection. This information included written material on the 2010 CPR guidelines, or the 2015 CPR guidelines when available.

**Acknowledgement/approval of the study**

Before the start of the recruitment, the trial will be submitted for approval to the Research Ethics Board of Medical University of Vienna.

**Insurance**

No insurance necessary.

**Confidentiality**

All subject names will be kept secret in the investigator's files. Subjects will be identified throughout documentation and evaluation by the number allotted to them during the study. The subjects will be told that all study findings will be stored and handled in strictest confidence.

**Documentation of study findings**

All findings collected during the study will be entered on the case report forms provided by the Medical Simulation and Emergency Management Research Group, Department of General Anaesthesia and Intensive Care Medicine. All entries in the case report forms will be made legibly in black ink. If corrections are made to entries in the case report form, the words or figures will be ringed and a single stroke drawn through them. The correct value will be entered beside the old entry and the correction will be dated and initialled. Incorrect entries must not be covered with correcting fluid, or obliterated, or made illegible in any way.

**Publication of study results**

The findings of this study will be published by the investigators in a scientific journal and presented at scientific meetings. The manuscript will be circulated to all co-investigators before submission.

**Authorship**

The principle investigator will be the first author, PD Dr. Bernhard Rössler. Senior-, and corresponding author will be Assoc. Prof. Dr. Karl Schebesta. Authorship will be granted for substantial contribution to the project. Supporting organisations will be acknowledged in the appropriate section of the final manuscript.

## References


- **Handley** AJ, Koster R, Monsieurs K, et al. European Resuscitation Council guidelines for resuscitation 2005. Section 2. Adult basic life support and use of automated external defibrillators.Resuscitation. 2005;67 Suppl 1:S7-23.

- **Koster** RW, Baubin MA, Bossaert LL, et al. European Resuscitation Council Guidelines for Resuscitation 2010 Section 2. Adult basic life support and use of automated external defibrillators. Resuscitation. 2010;81(10):1277-92.

- **Gräsner** JT, Bossaert L. Epidemiology and management of cardiac arrest: what registries are revealing. Best Pract Res Clin Anaesthesiol. 2013;27(3):293-306.

- **Valenzuela** TD, Bjerke HS, Clark LL, et al. Rapid defibrillation by nontraditional responders: the Casino Project. Acad Emerg Med 1998;5:414–5.

- **vanAlemAP**, Vrenken RH, de Vos R, et al. Use of automated external defibrillator by first responders in out of hospital cardiac arrest: prospective controlled trial. BMJ 2003;327:1312–7.

- **Wissenberg** M, Lippert FK, Folke F, et al. Association of national initiatives to improve cardiac arrest management with rates of bystander intervention and patient survival after out-of-hospital cardiac arrest. J Am Med Assoc 2013;310:1377–1384.

- **Müller** D, Agrawal R, Arntz HR. How sudden is sudden cardiac death? Circulation 2006;12,114:1146–50.
- **Swor** R, CPR training and CPR performance: do CPR-trained bystanders perform CPR? Acad Emerg Med 2006;13:596–601.

- **Savastano** S, Vanni V. Cardiopulmonary resuscitation in real life: The most frequent fears of lay rescuers. Resuscitation. 2011;82(5):568-71.

- **Rössler** B, Ziegler M, Hüpfl M, et al. Can a flowchart improve the quality of bystander cardiopulmonary resuscitation? Resuscitation. 2013;84(7):982-6.

- **Hüpfl** M, Selig HF, Nagele P. Chest-compression-only versus standard cardiopulmonary resuscitation: a meta-analysis. Lancet. 2010;376(9752):1552-7.

- **Lerner** EB, Rea TD, Bobrow BJ, et al. American Heart Association Emergency Cardiovascular Care Committee; Council on Cardiopulmonary, Critical Care, Perioperative and Resuscitation. Emergency medical service dispatch cardiopulmonary resuscitation prearrival instructions to improve survival from out-of-hospital cardiac arrest: a scientific statement from the American Heart Association. Circulation. 2012;125(4):648-55.

- **Heidenreich** JW, Bonner A, Sanders AB. Rescuer fatigue in the elderly: standard vs. hands-only CPR. J Emerg Med. 2012;42(1):88-92.

- **McDonald** CH, Heggie J, Jones CM, et al. Rescuer fatigue under the 2010 ERC guidelines, and its effect on cardiopulmonary resuscitation (CPR) performance. Emerg Med J. 2013;30(8):623-7.

- **Vadeboncoeur** T, Stolz U, Panchal A, et al. Chest compression depth and survival in out-of-hospital cardiac arrest. Resuscitation. 2014;85(2):182-8.

- **Stiell** IG, Brown SP, Nichol G, et al. What is the optimal chest compression depth during out-of-hospital cardiac arrest resuscitation of adult patients? Circulation. 2014;130(22):1962-70.

- **Idris** AH, Guffey D, Pepe PP, et al. Chest Compression Rates and Survival Following Out-of-Hospital Cardiac Arrest. Crit Care Med. 2015 [Epub ahead of print].

- **Monsieurs** KG, De Regge M, Vansteelandt K, et al. Excessive chest compression rate is associated with insufficient compression depth in prehospital cardiac arrest. Resuscitation. 2012;83(11):1319-23.

- **Stiell** IG, Brown SP, Christenson J, et al. What is the role of chest compression depth during out-of-hospital cardiac arrest resuscitation? Crit Care Med. 2012;40(4):1192-8.

- **Norman** G. Likert scales, levels of measurement and the "laws" of statistics. Adv Health Sci Educ Theory Pract 2010;15:625-32

- **Cariio** J, Perla R. Resolving the 50-year debate around using and misusing Likert scales. Med Educ 2008;42:1150-2.

- **R_Development_Core_Team**, R: A Language and Environment for Statistical Computing. 2009, R Foundation for Statistical Computing: Vienna, Austria.

- **Roessler** B, Fleischhackl R, Losert H, et al. Practical impact of the European Resuscitation Council's BLS algorithm 2005. Resuscitation. 2007;74(1):102-7.
